# FOXO3a-driven miRNA signatures suppresses VEGF-A/NRP1 signaling and breast cancer metastasis

**DOI:** 10.1038/s41388-020-01562-y

**Published:** 2020-12-01

**Authors:** Ying Song, Shanshan Zeng, Guopei Zheng, Danyang Chen, Pan Li, Mingqiang Yang, Kai Luo, Jiang Yin, Yixue Gu, Zhijie Zhang, Xiaoting Jia, Ni Qiu, Zhimin He, Hongsheng Li, Hao Liu

**Affiliations:** Affiliated Cancer Hospital and Institute of Guangzhou Medical University, Guangzhou Key Laboratory of “Translational Medicine on Malignant Tumor Treatment”, Guangzhou, 510095 PR China

**Keywords:** Breast cancer, Mechanisms of disease

## Abstract

Metastasis remains the major obstacle to improved survival for breast cancer patients. Downregulation of FOXO3a transcription factor in breast cancer is causally associated with the development of metastasis through poorly understood mechanisms. Here, we report that FOXO3a is functionally related to the inhibition of VEGF-A/NRP1 signaling and to the consequent suppression of breast cancer metastasis. We show that FOXO3a directly induces miR-29b-2 and miR-338 expression. Ectopic expression of miR-29b-2/miR-338 significantly suppresses EMT, migration/invasion, and in vivo metastasis of breast cancer. Moreover, we demonstrate that miR-29b-2 directly targets VEGF-A while miR-338 directly targets NRP1, and show that regulation of miR-29b-2 and miR-338 mediates the ability of FOXO3a to suppress VEGF-A/NRP1 signaling and breast cancer metastasis. Clinically, our results show that the FOXO3a-miR-29b-2/miR-338-VEGF-A/NRP1 axis is dysregulated and plays a critical role in disease progression in breast cancer. Collectively, our findings propose that FOXO3a functions as a metastasis suppressor, and define a novel signaling axis of FOXO3a-miRNA-VEGF-A/NRP1 in breast cancer, which might be potential therapeutic targets for breast cancer.

## Introduction

Breast cancer is the most common cancer diagnosed and the second leading cause of cancer-related deaths among women worldwide [1]. Despite progress in treating primary breast cancers with chemotherapy, radiation, surgery, and targeted therapies, metastasis and recurrence remain the major obstacles to improved survival for breast cancer patients [2]. Elucidating the molecular mechanisms of breast cancer metastasis/recurrence and searching for effective target therapies is a promising pathway for improving survival.

FOXO3a, a transcription factor of the Forkhead box O (FOXO) family, controls diverse biological processes by regulating the expression of gene involved in cell-cycle progression, apoptosis, differentiation, metabolism, or stress resistance [3]. FOXO3a is frequently downregulated in various cancers and functions as a tumor suppressor because it induces cell-cycle arrest and apoptosis [4–6]. Moreover, recent studies demonstrated that inactivation of FOXO3a can induce epithelial-to-mesenchymal transition (EMT) and subsequently promote cancer cell invasion and dissemination, indicating that FOXO3a can act as a potential biomarker for the prediction and therapy of cancer metastasis [7–11]. However, the biological function and exact mechanism of FOXO3a in regulating breast cancer metastasis are not completely understood.

Various previous studies identified some key signaling transduction cascades that are implicated in the progression, invasion, and metastasis of breast cancer [12]. Vascular endothelial growth factor (VEGF)-A, a main supervisor of angiogenesis, is a dimeric glycoprotein secreted by many kinds of cells, including cancer cells [13]. VEGF-A binds to and activates both VEGFR-1 and VEGFR-2, promoting angiogenesis [14]. Increased signaling through the VEGF-A/VEGFRs axis has been suggested to promote cancer cell invasiveness and metastasis and is associated with poor prognosis [15–17]. Specifically, neuropilin-1 (NRP1), a coreceptor of VEGF-A, forms complexes with VEGFRs to enhance the binding of VEGF-A to VEGFRs, thus promoting VEGF-A-mediated breast cancer cells EMT and metastasis [18–20]. It has been reported that VEGF-A is negatively regulated by FOXO3a, and FOXO3a-mediated repression of VEGF-A may be involved in transcriptional repression [21]. However, it is not clear whether other mechanisms may also account for this negative correlation between FOXO3a and VEGF-A. Specifically, no information is available as to whether microRNAs (miRNAs) play a role in the FOXO3a-mediated inhibition of VEGF-A/NRP1 signaling.

miRNAs are a class of small noncoding RNAs that regulate gene expression, either by inhibiting translation or by causing degradation through binding to the 3′-untranslated regions (UTRs) of target messenger RNAs [22]. Emerging evidence has revealed that miRNAs play key roles in multiple biological processes of cancer cells, including cancer cell proliferation, apoptosis, tumorigenesis, and metastasis [23]. Recently, several reports focused on the interaction of miRNAs and FOXO3a and confirmed the involvement of miRNAs in the FOXO3a-mediated regulation of cancer progression and metastasis [7, 24], suggesting that FOXO3a might use miRNAs to make cell fate decisions.

In the current study, we demonstrate that FOXO3a is downregulated in breast cancer, and overexpression of FOXO3a suppresses VEGF-A/NRP1 signaling and breast cancer invasion and metastasis. We further show that FOXO3a induces miR-29b/miR-338 expression by interacting with the miR-29b/miR-338 promoter and that miR-29b and miR-338 directly targets the VEGF-A and NRP1 3′-UTR, respectively. As a new member of the FOXO3a regulatory network, miR-29b/miR-338 may play a critical role in the posttranscriptional regulation of the VEGF-A/NRP1 axis and breast cancer metastasis. Therefore, our study suggests an important role of FOXO3a in inhibiting breast cancer metastasis and provides a previously undescribed mechanism of FOXO3a-mediated repression of VEGF-A/NRP1.

## Results

### FOXO3a inhibits invasion and metastasis of breast cancer

**Fig. 1:**
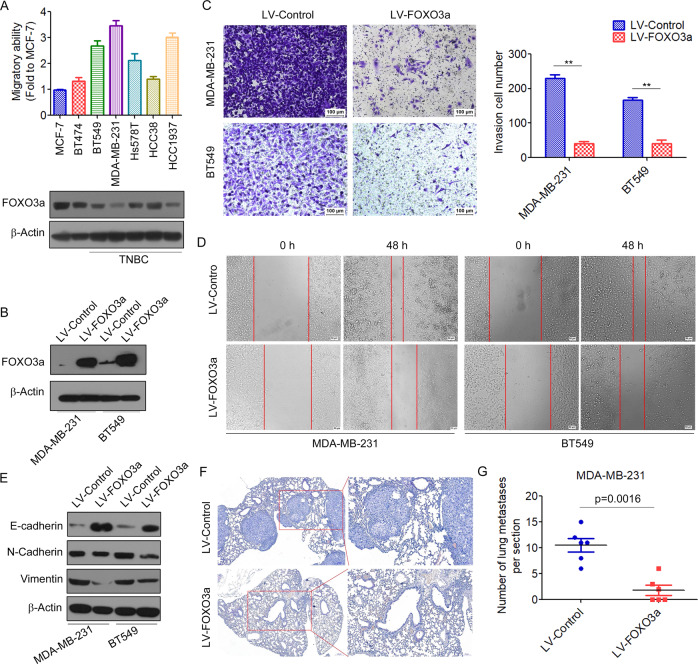
FOXO3a inhibits the invasion and metastasis of breast cancer cells. **A** The invasion ability of several breast cancer cell lines was analyzed by the transwell assay. Protein expression of FOXO3a in several breast cancer cell lines was analyzed by Western blot (below). **B** MDA-MB-231 and BT549 cells were transfected with LV-FOXO3a or LV-Control, and the expression of FOXO3a was analyzed by Western blot. The invasion and migration ability of the cells were determined by the transwell assay (**C**) and wound-healing assay (**D**). ^**^P < 0.01. **E** The expression of EMT markers was analyzed by Western blot. **F** MDA-MB-231 cells transfected with LV-FOXO3a or LV-control were injected into the tail veins of nude mice and followed over 6 weeks. Representative H&E staining of lung sections is shown. **G** The numbers of lung micrometastases per section were counted and analyzed.

We previously observed a negative correlation between FOXO3a expression and lymph node metastasis in breast cancer tissues (Supplementary Table 1) [25]. To determine the association between FOXO3a and tumor metastatic ability, we analyzed the protein expression of FOXO3a in several breast cancer cell lines. We found that the invasion ability of breast cancer cells was inversely correlated with FOXO3a expressions (Fig. 1A). We then performed a transwell assay to investigate the effects of FOXO3a on the invasive behaviors of breast cancer cells in vitro. The results demonstrated that overexpression of FOXO3a (Fig. 1B and Supplementary Fig. 1) significantly inhibited the invasion of MDA-MB-231 and BT549 cells (Fig. 1C). A wound-healing assay also demonstrated that overexpression of FOXO3a decreased the migration capacity of breast cancer cells (Fig. 1D). EMT, a highly conserved genetic program that enables epithelial tumor cells to migrate from the existing cell layer into surrounding tissues, is a key step in tumor metastasis [26]. We then investigated the effects of FOXO3a on the expression of EMT markers in breast cancer cells. We found that overexpression of FOXO3a inhibited EMT in breast cancer cells, which was evidenced by increased E-cadherin expression in conjunction with a concomitant decreased in the expression of mesenchymal markers vimentin and N-cadherin (Fig. 1E and Supplementary Fig. 2). To further investigate the inhibition of in vivo tumor metastasis by FOXO3a, we implanted MDA-MB-231/LV-FOXO3a cells that were stably expressing FOXO3a or control cells into nude mice through the lateral tail vein. Lung metastasis of breast cancer was apparent in mice injected with MDA-MB-231/LV-control cells. In contrast, few metastatic tumors were detected in mice injected with MDA-MB-231/LV-FOXO3a cells (Fig. 1F, G). Together, these results suggested that FOXO3a might function as a metastasis suppressor in breast cancer cells.

### FOXO3a inhibits VEGF-A/NRP1 signaling in breast cancer cells

**Fig. 2:**
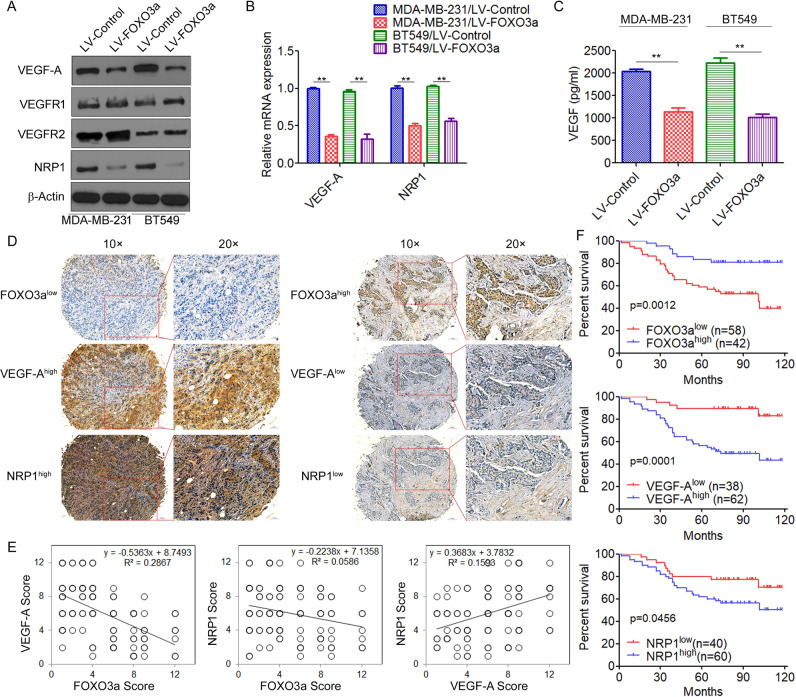
FOXO3a inhibits VEGF-A/NRP1 signaling in breast cancer cells. **A**–**C** MDA-MB-231 and BT549 cells were transfected with LV-FOXO3a or LV-Control; **A** the expression of VEGF-A, VEGFR-1, VEGFR-2, and NRP1 was analyzed by Western blot; **B** the expression of VEGF-A, and NRP1 was analyzed by qRT-PCR; **C** secreted VEGF was measured by ELISA. ^**^P < 0.01. **D** Photomicrographs showing representative IHC staining results for FOXO3a, VEGF-A, and NRP1 from TMA containing independent primary breast tumor samples. **E** FOXO3a, VEGF-A, and NRP1 expression in breast cancer tissue sections was quantitatively scored according to the percentage of positive cells and staining intensity as described in “Materials and methods.” Scatter plots showing an inverse correlation between the expression of FOXO3a and VEGF-A or NRP1 and a positive correlation between VEGF-A and NRP1. **F** Kaplan–Meier survival curves showing the association of FOXO3a, VEGF-A, and NRP1 expression and overall survival in patients with breast cancer.

The tumor microenvironment is increasingly recognized as an important contributor to tumor metastasis [27]. Cancer cells support their own progression by releasing multiple growth factors that interact with themselves directly through both autocrine and paracrine manners [28]. To gain further insight into how FOXO3a suppresses breast cancer metastasis, we examined the effect of FOXO3a on many microenvironmental genes, including TGF-β, TNF-α, EGF, FGF, PDGF, VEGF, and IGF, which have been implicated in promoting metastasis (Supplementary Fig. 3A). Importantly, we found that overexpression of FOXO3a significantly inhibited VEGF-A expression (Fig. 2A, B and Supplementary Fig. 3A) and secretion (Fig. 2C). Previous studies suggested that increased signaling through the VEGF-A/VEGFRs axis promotes cancer cell invasiveness and metastasis and is associated with poor prognosis [15–17]. We then investigated whether FOXO3a regulates VEGF-A receptor expression. We found that overexpression of FOXO3a had no effect on VEGFR-1 and VEGFR-2 expression, but significantly decreased the expression of NRP1 (Fig. 2A, B). In contrast, knockdown of FOXO3a by either of two short hairpin RNAs (shRNAs) significantly decreased the expression of VEGF-A and NRP1 (Supplementary Fig. 3B). Previous studies suggested that activation of PI3K/AKT and ERK signaling could induce phosphorylation and nuclear exclusion of FOXO3a, thereby inhibiting FOXO3a transcriptional activity [4–6]. We found that treatment with TIC10, a dual inhibitor of Akt and ERK [29], significantly increased the expression of FOXO3a, but decreased the expression of VEGF-A and NRP1 in MDA-MB-231 and BT549 cells (Supplementary Fig. 4).

We further compared FOXO3a, VEGF-A, and NRP1 expression in a tissue microarray containing 100 independent primary breast tumor samples by immunohistochemistry. We observed a significant inverse correlation between FOXO3a and VEGF-A/NRP1 expression levels, and a significant positive correlation between VEGF-A and NRP1 in the breast cancer tissue set (Fig. 2D, E). The correlation between FOXO3a and VEGF-A/NRP1 expression levels was further validated at the mRNA level in the cases from the cBioPortal database (Supplementary Fig. 5). Moreover, we found that high levels of VEGF-A and NRP1 were correlated with lymph node metastasis (Supplementary Table 2). Furthermore, we examined whether the levels of FOXO3a, VEGF-A, and NRP1 were associated with the survival of patients with breast cancer. Kaplan–Meier survival analyses revealed that patients with low FOXO3a expression had poorer overall survival than patients with high FOXO3a expression, whereas patients with high VEGF-A and NRP1 expression had poorer overall survival than patients with low VEGF-A or NRP1 expression (Fig. 2F). We next analyzed the correlation of FOXO3a, VEGF-A, and NRP1 expression with the prognosis of breast cancer patients with lymph node metastasis in the cases from the Kaplan–Meier plotter data set. Lower levels of FOXO3a and higher levels of VEGF-A or NRP1 were correlated with shorter survival in the lymph node metastasis positive subgroup (Supplementary Fig. 6A). However, no significant difference in prognosis was observed between lymph node metastasis negative breast cancer patients with high or low NRP1 expression (Supplementary Fig. 6B). Taken together, these findings indicated that FOXO3a negatively regulates VEGF-A/NRP1 and that dysregulated FOXO3a/VEGF-A/NRP1 signaling plays a critical role in disease progression in breast cancer.

### FOXO3a induces miR-29b and miR-338 expression in breast cancer cells

**Fig. 3:**
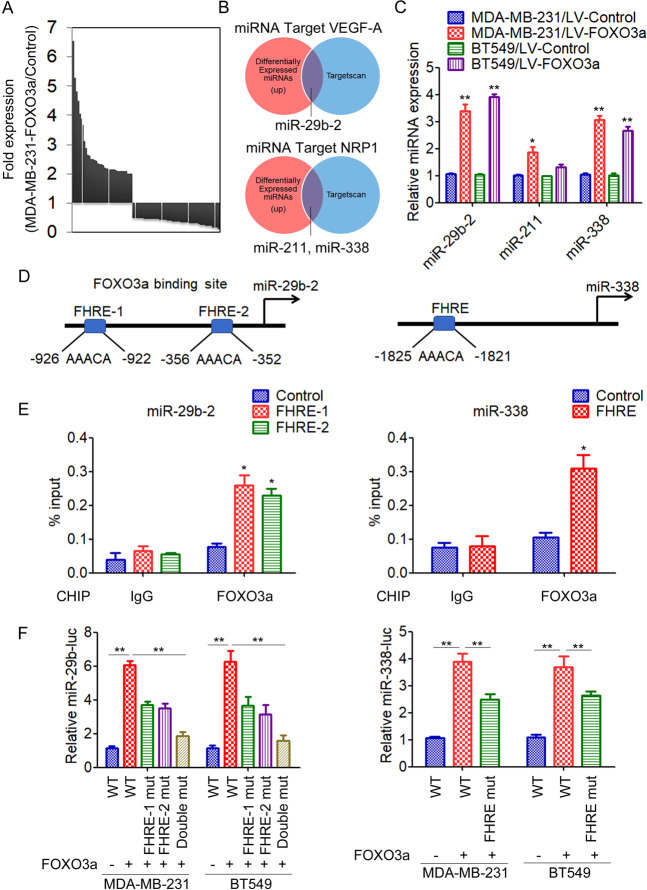
FOXO3a induces miR-29b-2 and miR-338 expression in breast cancer. **A** Unsupervised clustering analysis showed differentially expressed miRNAs in MDA-MB-231/FOXO3a cells compared with control cells. **B** Venn diagram analysis examined overlapping miRNAs with differentially expressed miRNAs and predicted miRNAs targeting VEGF-A/NRP1 via TargetScan. **C** Expression levels of miR-29b-2, miR-388, and miR-211 in MDA-MB-231/FOXO3a and BT549/FOXO3a cells were quantified by qRT-PCR. ^**^P < 0.01. **D** DNA sequence analysis revealed two FOXO3a-binding sites in the miR-29b-2 promoter region and a FOXO3a-binding site in the miR-338 promoter region. **E** A ChIP-qPCR assay was conducted to validate the binding of FOXO3a-binding sites to the promoter regions of miR-29b-2 and miR-388. ^*^P < 0.05. **F** Luciferase reporter was performed to confirm the regulation of the promoter regions of miR-29b-2 and miR-338 by FOXO3a. ^**^P < 0.01.

Previous studies suggested that FOXO3a directly binds to the VEGF-A promoter and inhibits its expression [21]. However, the NRP1 promoter lacked clear FOXO3a-binding sites, and FOXO3a did not decrease the activity of a luciferase reporter containing the NRP1 promoter (data not shown). We therefore speculated that FOXO3a might regulate VEGF-A/NRP1 indirectly through miRNAs. To identify differentially expressed miRNAs regulated by FOXO3a in breast cancer, we compared the miRNA profiles of the FOXO3a-overexpressing MDA-MB-231 cells and control cells by miRNA microarray analysis. Unsupervised clustering of significantly deregulated miRNAs is presented in Fig. 3A. We found that thirty-six miRNAs were significantly upregulated (greater than twofold) in MDA-MB-231/FOXO3a cells compared with MDA-MB-231/Control cells. In addition, forty-three miRNAs were downregulated (<50%) in MDA-MB-231/FOXO3a cells. The target mRNAs of differentially expressed miRNAs were predicted using TargetScan. We found that miR-29b-2, which targets VEGF-A, and miR-338 and miR-211, which targets NRP1 were upregulated in MDA231-FOXO3a cells (Fig. 3B). qRT-PCR analysis further confirmed that overexpression of FOXO3a significantly increased the expression level of miR-29b-2 and miR-338, but not miR-211, in both MDA-MB-231 and BT549 cells (Fig. 3C). In contrast, knockdown of FOXO3a significantly decreased the expression of miR-29b-2 and miR-338 in MCF-7 and BT474 cells (Supplementary Fig. 7). A sequence analysis of the promoter regions revealed that the miR-29b-2 promoter contains two FOXO3a-binding sites (FHRE-1, FHRE-2) and the miR-338 promoter contains one FOXO3a-binding site (FHRE) (Fig. 3D). To validate a direct binding of FOXO3a to the promoter region, we conducted a chromatin immunoprecipitation (ChIP)-qPCR assay using anti-FOXO3a antibody. The results demonstrated strong enrichment of FOXO3a in the miR-29-2 promoter regions and miR-338 promoter regions corresponding to the predicted FOXO3a-binding sites (Fig. 3E). To further demonstrate the regulation of the promoter regions of miR-29b-2 and miR-338 by FOXO3a, we performed a luciferase reporter assay. Our results showed that FOXO3a significantly increased the activity of both miR-29b-2 and miR-338 promoter reporter (Fig. 3F), and deleting the FHRE sites diminished FOXO3a-mediated reporter induction, demonstrating that these sites are necessary and functional (Fig. 3F).

### miR-29b-2 directly targets VEGF-A while miR-338 directly targets NRP1

**Fig. 4:**
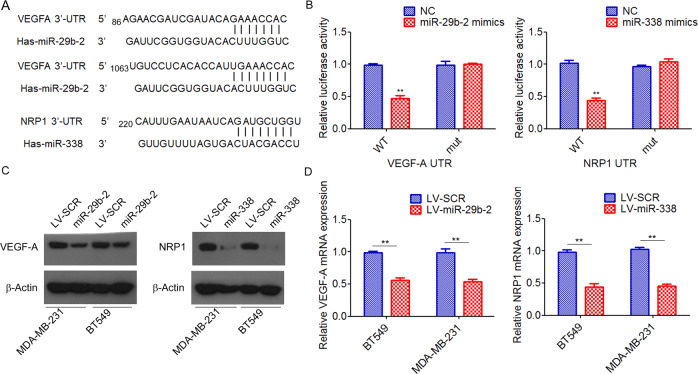
miR-29b-2 directly targets VEGF-A while miR-338 directly targets NRP1. **A** Schematic diagram showing the predicted miR-29b-2 site in the human VEGF-A mRNA 3′UTR and the predicted miR-338 site in the human NRP1 mRNA 3′UTR. **B** Relative luciferase activity was analyzed in HEK293T cells cotransfected with miRNA mimics and luciferase reporter vectors (pLuc-3′UTR). **C** The expression of VEGF-A and NRP1 in MDA-MB-231 and BT549 cells transfected with miR-29b-2 or miR-338 was measured by Western blot. **D** The expression of VEGF-A and NRP1 in MDA-MB-231 and BT549 cells transfected with miR-29b-2 or miR-338 was measured by qRT-PCR. ^**^P < 0.01.

TargetScan analyses revealed that the 3′-UTR of VEGF-A mRNA contains two highly conserved miR-29b-2 binding sites, whereas the 3′-UTR of NRP1 mRNA contains one highly conserved miR-338 binding site (Fig. 4A). We then tested whether VEGF-A and NRP1 are direct targets of miR-29b-2 and miR-338, respectively. Dual-luciferase reporter analysis showed that transfection of miR-29b-2 mimics significantly inhibited the activity of firefly luciferase that carried the wild-type but not mutant 3′UTR of VEGF-A (Fig. 4B). We also found that miR-338 mimics repressed the activity of firefly luciferase that carried the wild-type but not mutant 3′UTR of NRP1 (Fig. 4B). Furthermore, ectopic expression of miR-29b-2 in MDA-MB-231 and BT549 cells resulted in a marked decrease in VEGF-A mRNA and protein levels. Similarly, ectopic expression of miR-338 also significantly suppressed the mRNA and protein expression of NRP1 in breast cancer cells (Fig. 4C, D). Together, these data supported direct inhibition of VEGF-A by miR-29b-2 and NRP1 by miR-338.

### miR-29b-2 and miR-338 synergistically inhibit breast cancer invasion and metastasis

**Fig. 5:**
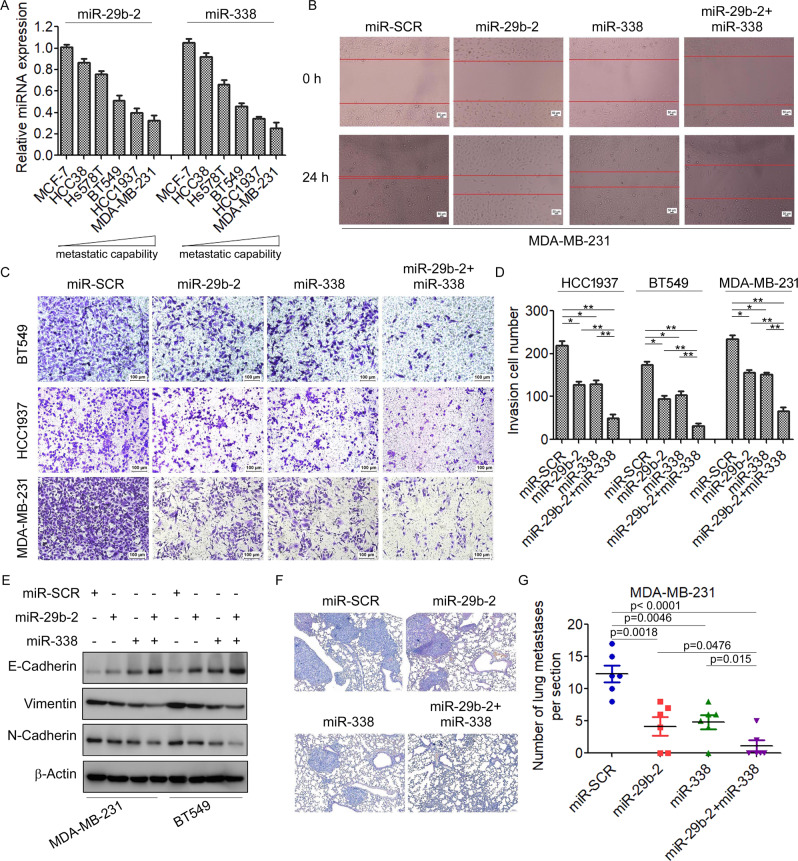
miR-29b-2 and miR-338 synergistically inhibit breast cancer invasion and metastasis. **A** The expression of miR-29b-2 and miR-338 in a data set of syngeneic cell lines with varying metastatic capabilities was measured by qRT-PCR. **B** The migratory capacity of MDA-MB-231 cells stably expressing miR-29b-2, miR-338, or miR-29b-2/miR-338 was determined by the wound-healing assay. **C**, **D** The invasion capacity of MDA-MB-231, BT549, and HCC1937 cells stably expressing miR-29b-2, miR-338, or miR-29b-2/miR-338 was determined by the transwell assay. ^*^P < 0.05, ^**^P < 0.01. **E** The expression of EMT markers in MDA-MB-231 and BT549 cells stably expressing miR-29b-2, miR-338, or miR-29b-2/miR-338 was measured by Western blot. **F** MDA-MB-231 cells stably expressing miR-29b-2, miR-338, or miR-29b-2/miR-338 were injected into the tail veins of nude mice and followed over 6 weeks. Representative H&E staining of lung sections is shown. **G** The numbers of lung micrometastases per section were counted and analyzed.

Using a data set of syngeneic cell lines with varying metastatic capabilities, we found that the expression levels of miR-29b-2 and miR-338 were lowest in cells with the highest metastatic capacities (Fig. 5A). The result suggested metastasis suppressor properties of miR-29b-2 and miR-338 in breast cancer cells. Therefore, functional analysis is required to clearly assign potential cancer-inhibition effects to one specific miR or the cooperation of both. To this end, we constructed lentiviral vectors for efficient and stable expression of miR-29b-2 or miR-338 or coexpression of miR-29b-2 and miR-338 in MDA-MB-231, BT549, and HCC1937 cells (Supplementary Fig. 8). A wound-healing assay demonstrated that stable expression of miR-29b-2 or miR-338 resulted in decreased migration and invasion. Importantly, coexpression of miR-29b-2 and miR-338 significantly decreased migration and invasion compared to those observed with stable expression of miR-29b-2 or miR-338 alone (Fig. 5B). Moreover, the transwell assay results also showed that overexpression of miR-29b-2/miR-338 significantly inhibited the migration and invasion of MDA-MB-231, BT549, and HCC1937 cells (Fig. 5C, D). In addition, we analyzed the impact of miR-29b-2/miR-338 on the EMT. In accordance with the effects of FOXO3a on EMT (Fig. 1), stable expression of miR-29b-2/miR-338 resulted in an increase in E‐cadherin but decreased levels of vimentin and N-cadherin (Fig. 5E). To further investigate the inhibition of in vivo tumor metastasis by miR-29b/miR-338, we implanted MDA-MB-231/miR-29b-2, MDA-MB-231/miR-338, or MDA-MB-231/miR-29b-2/miR-338 cells into nude mice through the lateral tail vein. We found that stable expression of miR-29b-2/miR-338 significantly decreased lung metastasis of breast cancer compared with that in control cells (Fig. 5F, G). Taken together, these results suggested that miR-29b-2 and miR-338 synergistically inhibits breast cancer invasion and metastasis.

### Regulation of miR-29b-2 and miR-338 mediates the ability of FOXO3a to suppress VEGF-A/NRP1 signaling and breast cancer metastasis

**Fig. 6:**
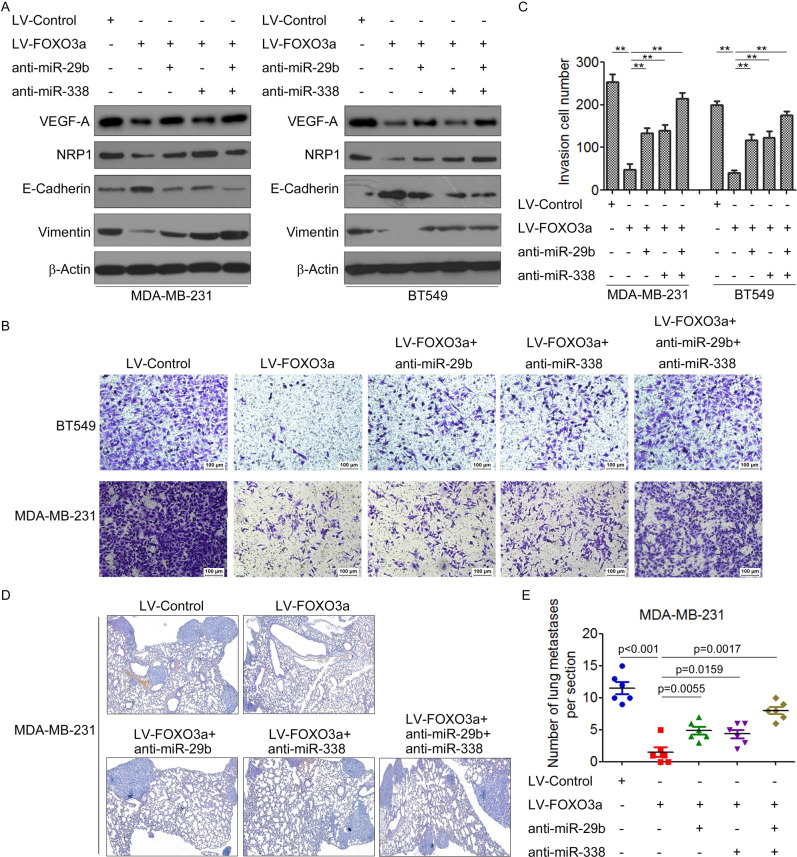
Regulation of miR-29b-2 and miR-338 mediates the ability of FOXO3a to suppress VEGF-A/NRP1 signaling and breast cancer metastasis. **A** MDA-MB-231 and BT549 cells were transfected with FOXO3a alone or FOXO3a combined with anti-miR-29b-2 or/and anti-miR-338, and the expression of VEGF-A, NRP1, E-cadherin, and vimentin was measured by Western blot. **B, C** The invasion capability of MDA-MB-231 and BT549 cells transfected with FOXO3a alone or FOXO3a combined with miRNA-29b-2 and miR-338 was determined by the transwell assay. ^**^P < 0.01. **D** MDA-MB-231 cells were transfected with FOXO3a alone or FOXO3a combined with miRNA, and then injected into the tail veins of nude mice and followed over 6 weeks. Representative H&E staining of lung sections is shown. **E** The numbers of lung macrometastases per section were counted and analyzed.

To demonstrate the role of miR-29b-2 and miR-338 in the FOXO3a-mediated repression of VEGF-A/NRP1 and breast cancer metastasis, we determined whether the FOXO3a-mediated repression of VEGF-A/NRP1 can be reversed by knockdown of miR-29b-2 and miR-338. Indeed, although overexpression of FOXO3a reduced the VEGF-A level, this reduction was blocked by anti-miR-29b-2 in MDA-MB-231/FOXO3a and BT549/FOXO3a cells (Fig. 6A and Supplementary Fig. 9). Similarly, knockdown of miR-338 abrogated the inhibitory effect of FOXO3a on NRP1 (Fig. 6A). We then investigated whether FOXO3a-mediated metastasis suppression requires miR-29b and miR-338. We found that loss of miR-29-2 or miR-338 in MDA-MB-231/FOXO3a and BT549/FOXO3a cells caused a mesenchymal morphology accompanied by an increase in the levels of vimentin but decrease in the level of E-cadherin (Fig. 6A), whereas transfected with miR-29b-2 or/and miR-338 reversed the effect of FOXO3a shRNA on the expression of E-cadherin and vimentin in MCF-7 cells (Supplementary Fig. 10). Furthermore, overexpression of FOXO3a inhibited cell invasion, and concomitant loss of miR-29b-2 and miR-338 abrogated the effects of FOXO3a, resulting in increased cell invasion (Fig. 6B, C). Significantly, we found that knockdown of miR-29b-2 and miR-338 reversed FOXO3a-mediated suppression of in vivo tumor metastasis (Fig. 6D, E). Consistently, overexpression of FOXO3a decreased the expression of VEGF-A and NRP1 but increased the expression of E-cadherin, whereas inhibition of miR-29b-2 and miR-338 reversed the effect of FOXO3a on the expression of VEGF-A, NRP1, and E-cadherin in the metastatic lesions (Supplementary Fig. 11). Together, our results demonstrated that miR-29b-2 and miR-338 are important downstream factors of FOXO3a that control VEGF-A/NRP1 signaling, ultimately leading to metastasis suppression.

### FOXO3a-miRNA-VEGF-A/NRP1 signaling is dysregulated in human breast cancer

**Fig. 7:**
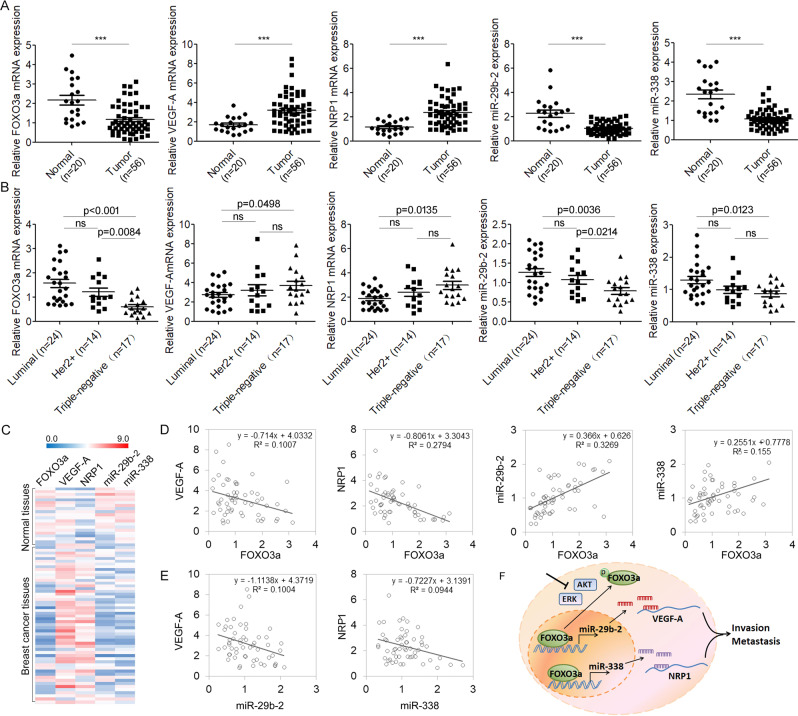
FOXO3a-miRNA-VEGF-A/NRP1 signaling is dysregulated in human breast cancer. **A** The expression of FOXO3a, VEGF-A, NRP1, miR-29b-2, and miR-338 expression in 56 breast cancer tissues and 20 normal tissues was measured by qRT-PCR. ^***^P < 0.001. **B** FOXO3a, VEGF-A, NRP1, miR-29b-2, and miR-338 expression patterns were analyzed in breast cancer tissues with Luminal, HER2+ and triple-negative types. **C** Heatmap diagram of differential expression of FOXO3a, VEGF-A, NRP1, miR-29b-2, and miR-338 in normal and breast cancer tissues. The color scale at the top illustrates the relative expression level of the mRNAs or miRNAs. **D** Scatter plots represented a negative correlation between FOXO3a and VEGF-A or NRP1 levels and a positive correlation between FOXO3a and miR-29b or miR-338 levels. **E** Scatter plots represented a negative correlation between VEGF-A and miR-29b-2 levels or between NRP1 and miR-338 levels. **F** A network model of FOXO3a-miRNA-VEGF-A/NRP1 signaling is proposed.

To further confirm that FOXO3a-miRNA-VEGF-A/NRP1 signaling is dysregulated in human breast cancer, expression levels of FOXO3a, miR-29b-2, miR-338, VEGF-A, and NRP1 were quantified in total RNA derived from 34 breast cancer tissues and 20 normal breast tissues. We found that the mRNA expression levels of FOXO3a, miR-29b-2, and miR-338 were significantly decreased in tumor tissues, whereas those of VEGF-A and NRP1 were significantly increased in tumor tissues (Fig. 7A). Next, using mRNA expression data from 34 primary patient tumor samples for which hormone receptor and HER2 expression data were available, we found that VEGF-A and NRP1 mRNA expression levels were significantly elevated in triple-negative tumors compared with hormone receptor-positive or HER2-positive tumors (Fig. 7B). In contrast, FOXO3a, miR-29b, and miR-338 had the lowest expression levels in triple-negative tumors. Furthermore, our results generally showed a negative correlation between FOXO3a and VEGF-A or NRP1 levels, a positive correlation between FOXO3a and miR-29b or miR-338 levels (Fig. 7C, D), and a negative correlation between VEGF-A and miR-29b levels or between NRP1 and miR-338 levels (Fig. 7E). Taken together, our findings suggested that FOXO3a-miRNA-VEGF-A/NRP1 signaling is dysregulated and plays a critical role in disease progression of breast cancer.

## Discussion

Metastasis is the process by which tumor cells spread from the primary tumor to distant organs and form secondary tumors [30]. Metastatic dissemination of breast cancer cells remains the major obstacle to effective therapy [31]. The loss of function of metastasis suppressor genes is a major rate-limiting step in breast cancer progression that prevents the formation of new colonies at distal sites [32]. Several lines of evidence demonstrated that downregulation of FOXO3a affects phenotypes such as cell proliferation, EMT, and stemness that contribute to breast cancer progression and poor response to therapies [10, 25, 33, 34]. In the current study, our findings demonstrated that FOXO3a expression is inversely correlated with the migration ability of breast cancer cells and overexpression of FOXO3a suppresses breast cancer invasion and metastasis. In addition, we found that FOXO3a-driven miR-29b/miR-338 expression plays a critical role in the posttranscriptional regulation of VEGF-A/NRP1 signaling and breast cancer metastasis.

The role of FOXO3a in the regulation of cancer cell invasion and metastasis has been investigated in different cellular models [7–10, 35]. For example, FOXO3 downregulation increased the expression of Twist1 and cell motility in urothelial cancer cells [36]. FOXO3a expression is also decreased in renal cell carcinoma and is associated with metastasis-free survival of patients [9]. Our previous studies also demonstrated that FOXO3a modulates WNT/β-catenin signaling and suppresses EMT in prostate cancer cells [7]. To date, the role of FOXO3a in breast cancer metastasis remains controversial. Most studies have defined FOXO3a proteins as a metastasis suppressor, as it induces E-cadherin expression and represses EMT-inducing transcription factors, which in turn reversed the invasive phenotype of breast cancer cells [10]. In contrary, FOXO3a was demonstrated as a MMP-9 and MMP-13 inducer promoting the invasion of HeLa and MDA-MB-435 cell lines [37]. In this study, we found that overexpression of FOXO3a significantly suppressed the migration and invasion of breast cancer cells in vitro. Moreover, the antimetastatic function of FOXO3a was further confirmed using a nude mouse xenograft model. Therefore, we propose that FOXO3a as a metastasis suppressor and that loss of function of FOXO3a might promote breast cancer progression. Interestingly, a recent study suggested that either activation or loss of FOXO3a function suppressed breast cancer growth and metastasis. A potential explanation might be that metastasis is delayed in both situations because primary tumor growth is also delayed and a tumor needs to reach a certain size or stage before it can metastasize [38]. Moreover, Stephan et al. suggested that high nuclear β-catenin content might subvert FOXO3a to promote metastasis by enhancing the expression of many FOXO3a target genes that are involved in cytoskeleton remodeling and motility in colon cancer [39]. It would therefore be interesting to study the role of FOXO3a in suppressing or supporting breast cancer cells with diverse biological properties and various amounts of nuclear β-catenin or some factors.

Our study provides evidence that the antimetastatic effect of FOXO3a could be partially caused by VEGF-A/NRP1 repression. VEGF-A is one of the major inducers of angiogenesis and is highly correlated with tumor progression, invasion, and metastasis in breast cancer [40]. A previous study demonstrated that treatment with Lapatinib resulted in nuclear translocation and activation of FOXO3a, followed by a reduction in VEGF expression in BT474 and SKBR3 cells [21]. Similarly, we also found that overexpression of FOXO3a significantly inhibits VEGF-A expression and secretion, as well as the expression of its coreceptor NRP1. Moreover, we observed a significant inverse correlation between FOXO3a and VEGF-A/NRP1 expression levels, and a significant positive correlation between VEGF-A and NRP1 in the breast cancer tissues. Lower levels of FOXO3a, and higher levels of VEGF-A or NRP1 were correlated with shorter survival in the lymph node metastasis positive subgroup. Thus, our results indicated that FOXO3a is a negative regulator of VEGF-A/NRP1 and that dysregulated FOXO3a/VEGF-A/NRP1 signaling contributes to disease progression of breast cancer.

As a master regulator of gene expression, FOXO3a has been shown to directly or indirectly regulate numerous protein-coding genes [3]. It has been reported that FOXO3a-mediated repression of VEGF-A might be involved in transcriptional repression [21]. However, it is not clear whether other mechanisms may also account for the negative correlation between FOXO3a and VEGF-A. An important strength of our current work is that we define the role of miR-29b in the posttranscriptional regulation of VEGF-A by FOXO3a and suggest that, as a new member of the FOXO3a regulatory network, miR-29b provides a direct link between FOXO3a and VEGF-A in this gene regulatory network. To date, only a very limited number of miRNAs has been shown to be direct targets of FOXO3a. For example, miR-622 overexpression mediated by FOXO3a was shown to repress the invasiveness of lung tumor cells by inhibition of HIF-1α mediated by ERK inactivation in U0126-treated A549 cells [41]. Our previous study also demonstrated that miR-34b/c is directly regulated by FOXO3a and that FOXO3a-mediated transactivation of miR-34b/c inhibits WNT/β-catenin signaling and suppresses β-catenin-dependent EMT in prostate cancer [7]. Therefore, the identification of miR-29b/miR-338 as a direct FOXO3a target in this study expands the repertoire of FOXO3a-regulated genes. miR-29b targets a network of pro-metastatic regulators involved in EMT, collagen remodeling, and proteolysis, including Snail, MMPs, Mcl-1, COL1A1, TGF-β, VEGF, and PDGF [42, 43]. As about miR-338, it also exerts tumor suppressive effects by targeting a variety of oncogenes and upregulating tumor suppressors [44, 45]. In accordance with these previous studies, we found that miR-29b and miR-338 activated by FOXO3a directly targets VEGF-A and NRP1, respectively. Moreover, miR-29b cooperated with miR-338 to inhibit breast cancer invasion and metastasis in both in vitro and in vivo models. In particular, we found that the expression of miR-29b and miR-338 was significantly reduced in human breast cancer tissues. Furthermore, our results generally showed a negative correlation between FOXO3a and VEGF-A or NRP1 levels, a positive correlation between FOXO3a and miR-29b or miR-338 levels, and a negative correlation between VEGF-A and miR-29b levels or between NRP1 and miR-338 levels. Therefore, we propose that a reduction in miR-29b/miR-338 expression elicited by FOXO3a inactivation results in alleviation of miR-29b/miR-338-mediated targeting of VEGF-A/NRP1, thus contributing to the aggressive behavior of breast cancers.

In conclusion, our observations support the hypothesis that FOXO3a is a metastasis suppressor that performs its effect by inhibiting EMT, invasion, and metastatic progression of breast cancers. Our study reveals a novel mechanistic insight for breast cancer metastasis mediated by the FOXO3a-miRNA-VEGF/NRP1 signaling axis and highlights the potential of targeting FOXO3a-miR-29b/miR-338 as a novel therapeutic strategy for breast cancer (Fig. 7F).

## Materials and methods

### Cell culture

The human breast cancer cell line MCF-7, BT474, BT549, MDA-MB-231, Hs578T, HCC38, and HCC1937 were obtained from the American Type Culture Collection and authenticated by short tandem repeat analysis every 6 months after used in our laboratory. All cell lines were maintained with Dulbecco’s Modified Eagle Medium (HyClone, Logan, UT, USA) containing 10% fetal bovine serum and 1% penicillin/streptomycin in a humidified incubator of 5% CO_2_ at 37 °C.

### Patients and specimens

Primary tumor specimens were obtained from 100 patients diagnosed with breast cancer who underwent complete resection in the Affiliated Tumor Hospital of Guangzhou Medical University between 2004 and 2008. Follow-up information was obtained from review of the patients’ medical record. Furthermore, 56 of fresh primary breast cancer tissues and 20 of normal breast tissues used in this study were collected from the Affiliated Tumor Hospital of Guangzhou Medical University. Written informed consent was obtained from all study participants. This study was approved by the Ethics Committee of Guangzhou Medical University and written informed consent was provided by all patients based on the Declaration of Helsinki.

### Lentivirus production and transduction

FOXO3a ORF was cloned into the lentiviral vector GV358 (GENECHEM, Shanghai, China). FOXO3a shRNAs or Control shRNA sequences were cloned into the EGFP-labeled lentiviral vector GV248 (GENECHEM). The target sequences selected are shown in Supplementary Table 3. Lentivirus was generated using the packaging plasmids pHelper 1.0 and pHelper 2.0 (GENECHEM). Recombinant lentiviruses containing hsa-mir-29b-2, hsa-mir-338, or scrambled sequences were purchased from GENECHEM. Anti-miR-29b-2 and anti-miR-338 were obtained from GeneCopoeia Inc. (Guangzhou, China). Lentiviruses production was added to cells with Polybrene, and stably transduced cells were selected in puromycin for at least 5 days.

### MiRNA microarray analysis

Total RNA, containing miRNA, was extracted from MDA-MB-231/FOXO3a cells and MDA-MB-231/control cells using TRIzol reagent (Invitrogen, Carlsbad, CA) according to the manufacturer’s instructions. The miRNA expression profiles were generated by using the Affymetrix GeneChip miRNA Array v. 4.0 (Affymetrix). Briefly, the flashTag Biotin RNA Labeling Kit (Affymetrix) was used to label 1 μg of total RNA, followed by the hybridization overnight according to the manufacturer’s instructions. Images were scanned using the GeneChip Scanner 3000 and image analysis was done with the GeneChip Operating Software.

### Real-time RT-PCR

For mRNA detection, total RNAs were extracted using the E.Z.N.A.^®^ HP Total RNA Kit (Omega Bio-tek, Doraville, GA, USA), followed by reverse-transcription using the PrimeScript^®^ RT reagent Kit (TakaRa, Shiga, Japan), and quantitative PCR using the SYBR Green qRT-PCR according to the manufacturer’s instructions (Applied Biosystems). The primer pairs used are shown in Supplementary Table 4.

For miRNA detection, the reverse transcribed cDNA was synthesized with the All-in-One™ miRNA First-Strand cDNA Synthesis Kit (GeneCopoeia, Rockville, MD, USA). miR-29b-2 and miR-338 expression was determined with the All-in-One™ miRNA qRT-PCR Detection Kit (GeneCopoeia, Rockville, MD, USA).

### Western blot

Western blot analysis was performed as previously described. Briefly, total protein was isolated using RIPA buffer (Beyotime Biotechnology, China) in the presence of protease inhibitor cocktail. The protein concentration of lysates was measured using a BCA Protein Assay Kit (Thermo Scientific). Equivalent amounts of protein were separated by SDS-PAGE and transferred to a PVDF membrane. The membranes were subsequently blocked in 5% nonfat milk and then incubated with primary antibodies. After extensive washing, immunoreactivity was detected using secondary antibody-conjugated horseradish peroxidase. Signals were captured using ECL and x-ray film. The antibodies used for Western blotting assays are shown in Supplementary Table 5.

### Animal studies

All animal work was performed in accordance with protocols approved by the Animal Experimentation Ethics Committee of Guangzhou medical University. 1 × 10^6^ cells were injected into the tail vein of 6-week-old female nude mice (*N* = 6 per group). After 6 weeks, mice were sacrificed. Their lungs were fixed in 4% paraformaldehyde, paraffin-embedded and sliced. Lung sections were stained by hematoxylin and eosin (H&E) and immunohistochemical assay. The numbers of micrometastases in the lungs per tissue section in individual mice were determined from morphological observation of H&E-stained sections.

Wound-healing assay, transwell assay, ELISA assay, ChIP, Dual-luciferase reporter assays, and immunohistochemical assay are described in the Supplementary Experimental Procedure.

### Statistical analysis

Statistical analyses were conducted using the SPSS16.0 software. Comparisons between groups were analyzed by the *t* test and *χ*^2^ test. Overall survival curves were plotted according to the Kaplan–Meier method with the log-rank test applied for comparison. Survival was measured from the day of surgery. Variables with values of *P* < 0.05 by univariate analysis were used in subsequent multivariate analysis based on the Cox proportional hazards model. The differences were considered statistically significant at *P* < 0.05.

## Supplementary information

Supplementary Figure 1-11

Supplementary Table 1-5

Supplementary Experimental Procedures
