# Supplementary material for: FOXO3a-driven miRNA signatures suppresses VEGF-A/NRP1 signaling and breast cancer metastasis
Source: Oncogene. 2020 Dec 1;40(4):777–90. doi: 10.1038/s41388-020-01562-y (PMC7843418; doi:10.1038/s41388-020-01562-y)
Supplement: Supplementary file 1 — Supplementary Figure 1-11 [file 41388_2020_1562_MOESM1_ESM.docx]

**Supplementary Figure 1-11**

**
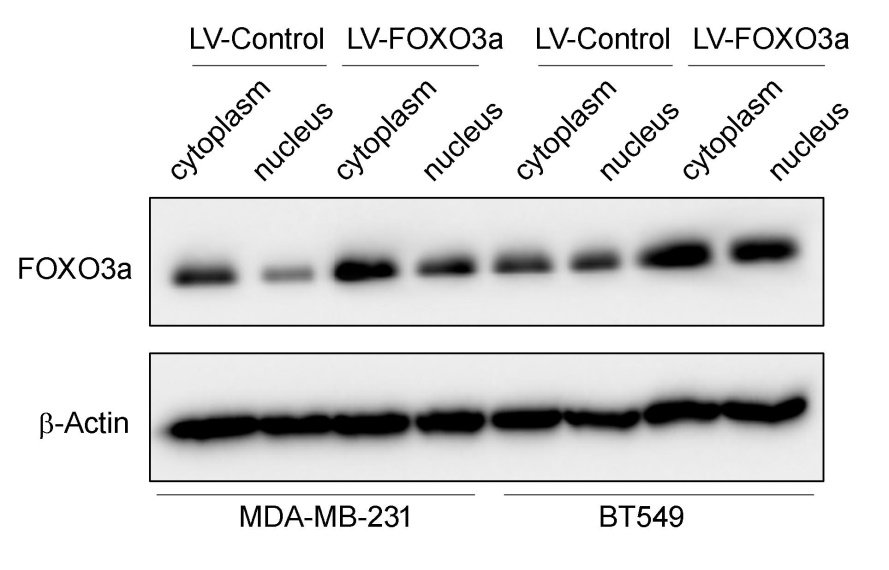
**

**Supplementary Fig. 1.** MDA-MB-231 and BT549 cells were transfected with LV-FOXO3a or LV-Control, Nuclear and cytoplasmic lysates were prepared, changes in FOXO3a protein level were analyzed by Western blot.

**
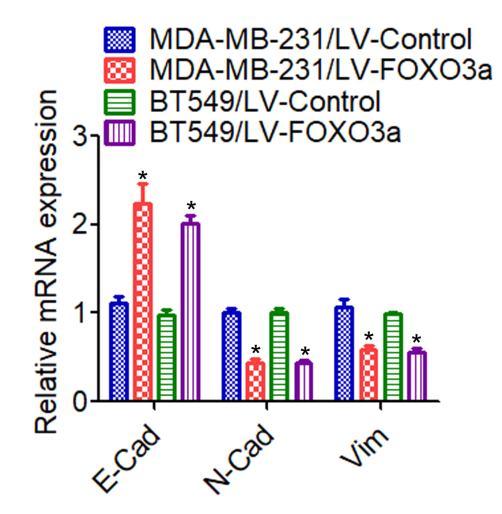
**

**Supplementary Fig. 2.** Expression of EMT markers in MDA-MB-231 and BT549 cells transfected with LV-FOXO3a or LV-Control were measured by qRT-PCR (^*^*P* < 0.05).

**
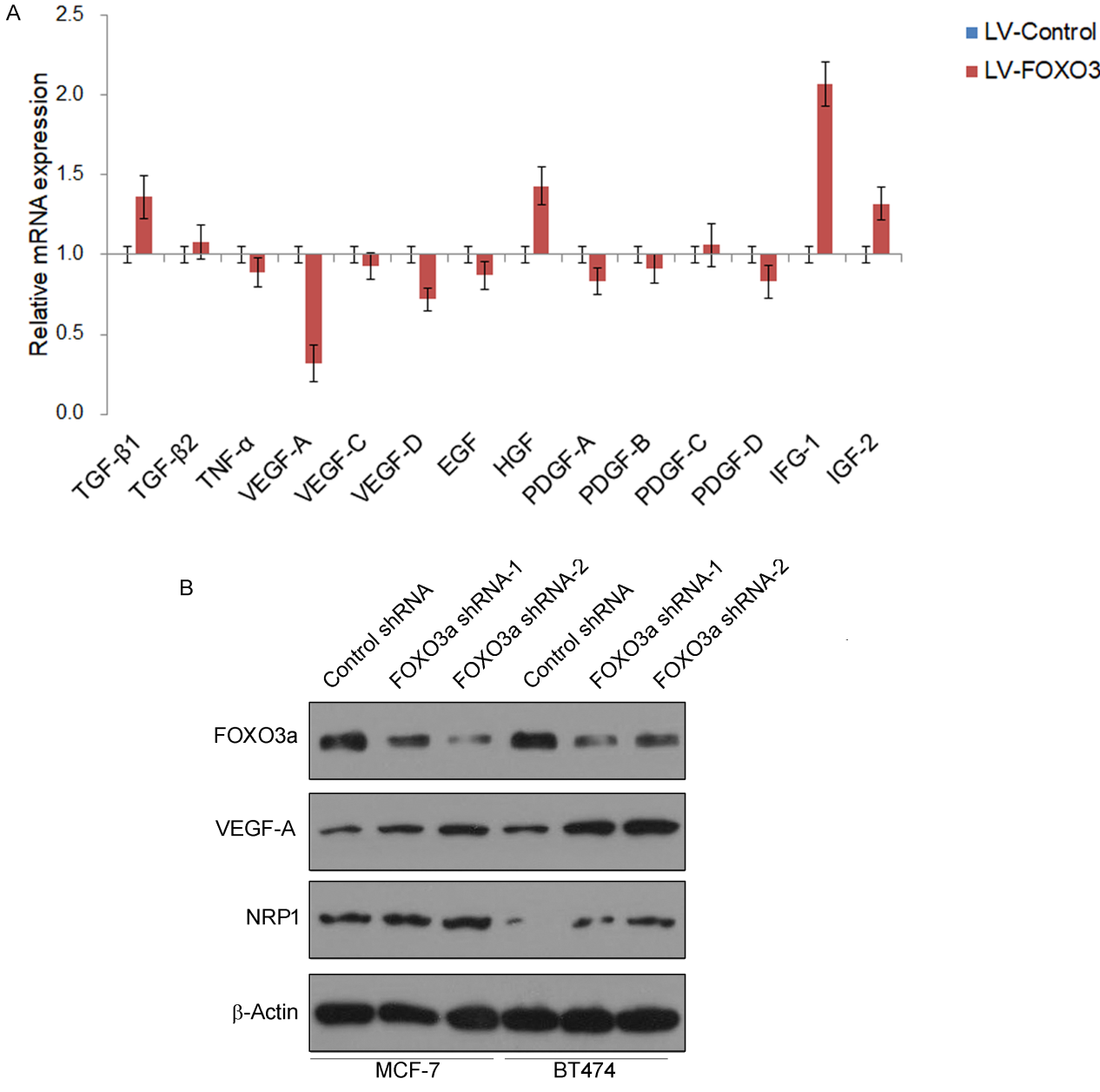
**

**Supplementary Fig. 3. (A)** Expression of microenvironmental genes (TGF-β, TNF-α, EGF, FGF, PDGF, VEGF and IGF) in MDA-MB-231 cells transfected with LV-FOXO3a or LV-Control were measured by qRT-PCR. (B) MCF-7 and BT474 cells were transfected with FOXO3a shRNA, the expression of FOXO3a, VEGF-A and NRP1 were measured by Western blot analysis.


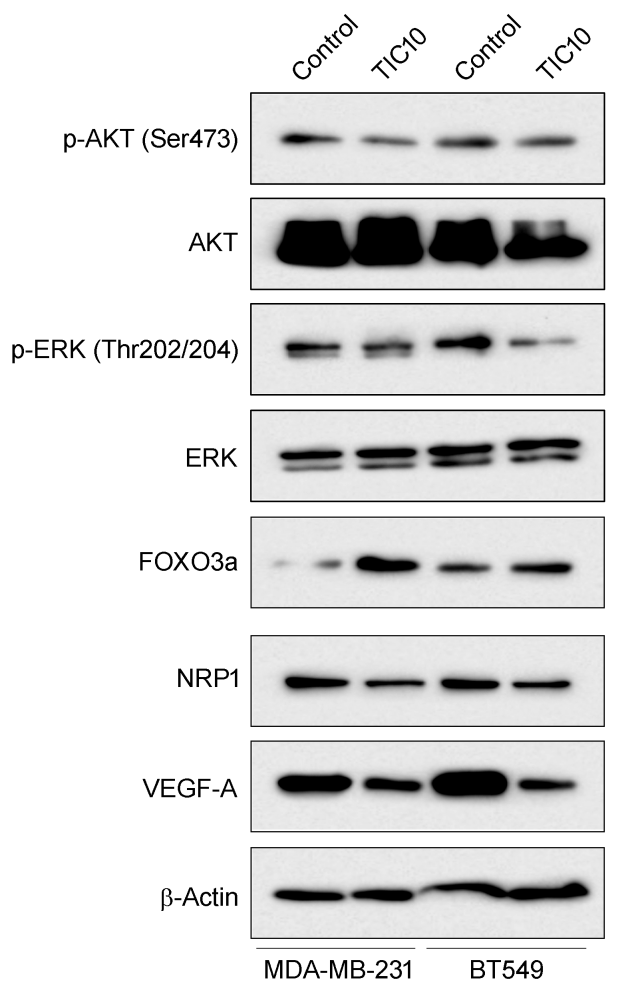


**Supplementary Fig. 4.** MDA-MB-231 and BT549 cells were treated with 10 μM TIC10 for 48 h, the expression of indicated protein were measured by Western blot analysis.

**
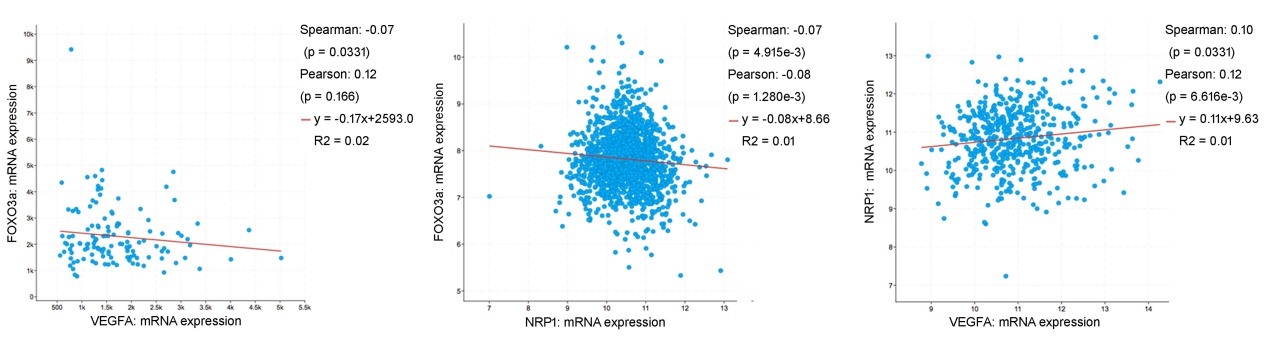
**

**Supplementary Fig. 5. T**he correlation between FOXO3a and VEGF-A/NRP1 expression levels in breast cancer was identified from cBioPortal database (http://cbioportal.org).


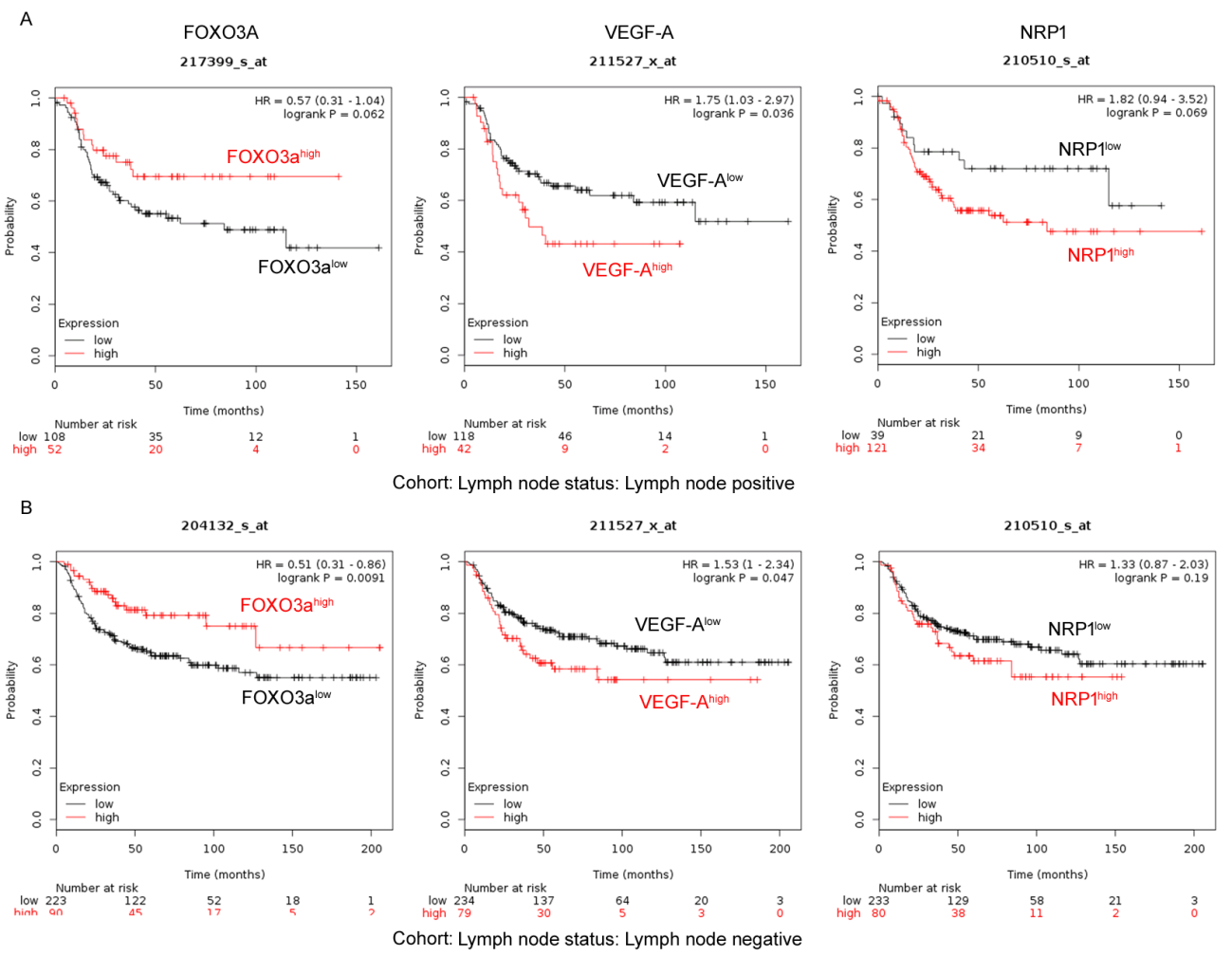


**Supplementary Fig. 6.** Kaplan–Meier OS curves (http://kmplot.com/analysis/) of breast cancer patients relative to different expression levels of FOXO3a (probe 217399_x_at), VEGF-A (probe 211527_x_at) and NRP1 (probe 210510_x_at).


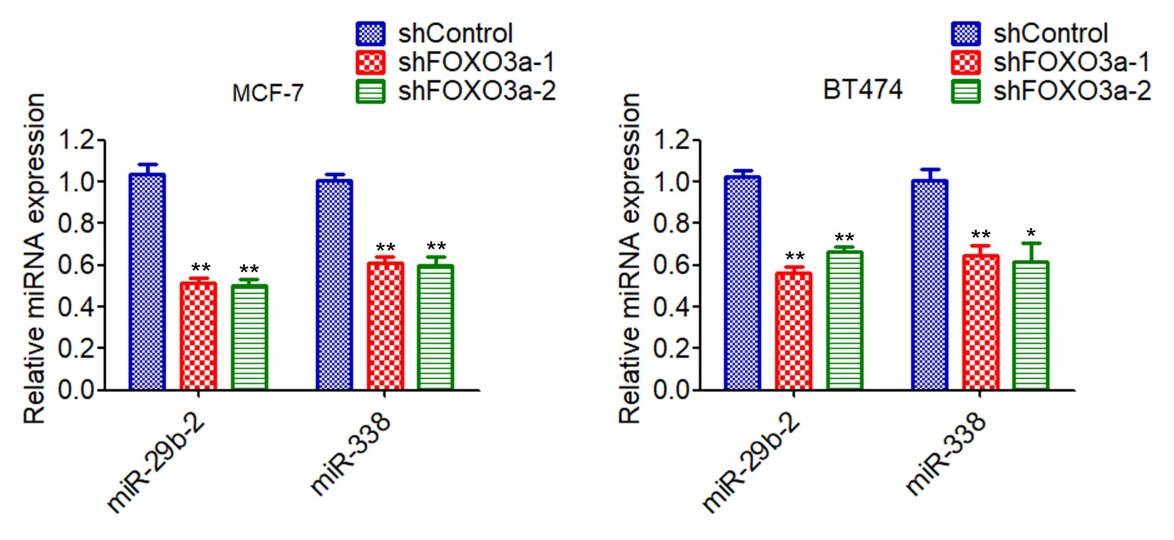


**Supplementary Fig. 7.** MCF-7 and BT474 cells were transfected with FOXO3a shRNA, the expression of miR-29b-2 and miR-388 were tested by qRT-PCR. ^*^*P* < 0.05, ^**^*P* < 0.01.


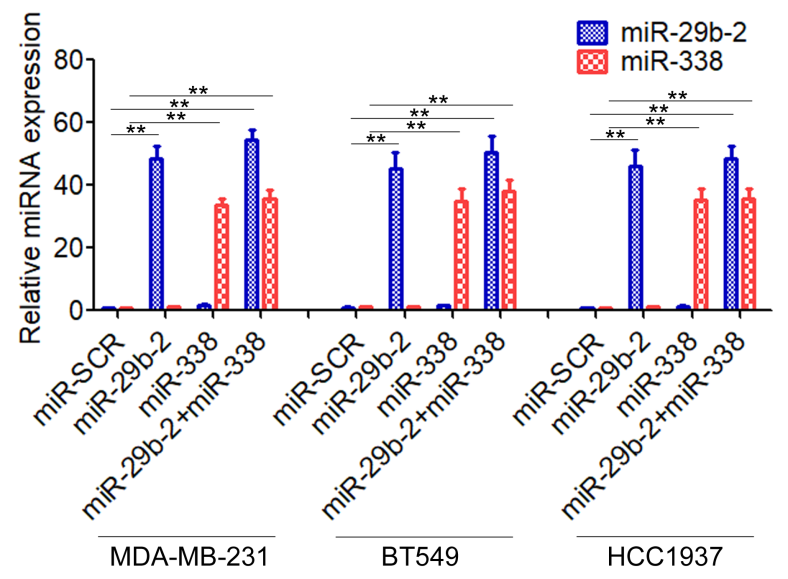


**Supplementary Fig. 8.** MDA-MB-231, BT549 and HCC1937 cells were transfected with miR-29b-2 or/and miR-338, expression of miR-29b-2 and miR-338 were measured by qRT-PCR (^**^*P* < 0.01).


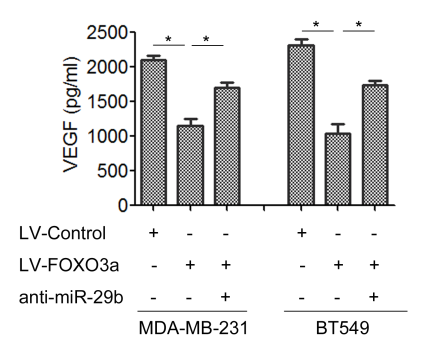


**Supplementary Fig. 9.** MDA-MB-231/FOXO3a and BT549/FOXO3a cells were transfected with miR-29b-2, Secreted VEGF protein concentrations were measured by ELISA (^*^*P* < 0.05).


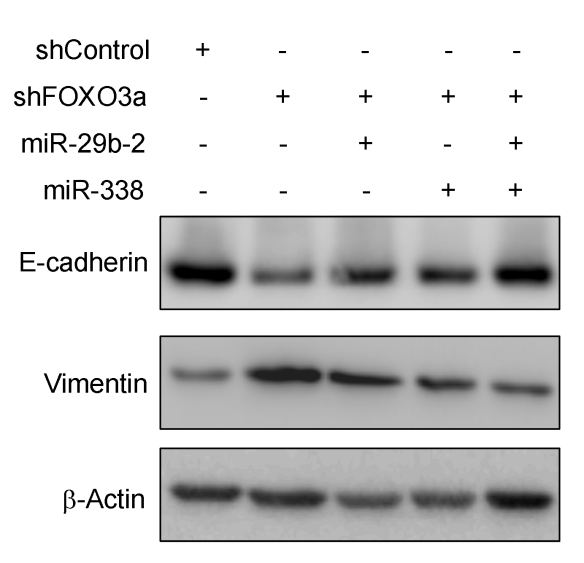


**Supplementary Fig. 10.** MCF-7 cells transfected with FOXO3a shRNA alone or FOXO3a shRNA combined with miR29b-2 or/and miR-338, the expression of E-cadherin and Vimentin were measured by Western blot.


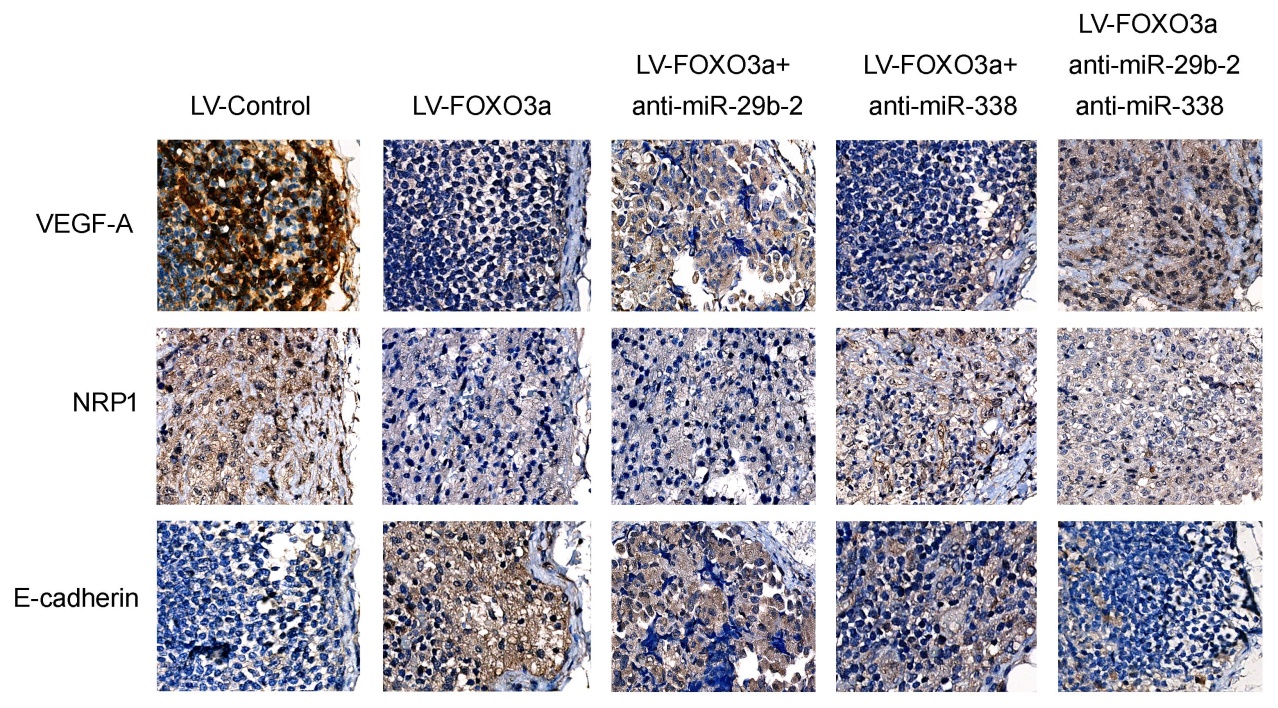


**Supplementary Fig. 11.** Photomicrographs showed representative IHC staining results of VEGF-A, NRP1 and E-cadherin in the metastatic lesions.
