# Supplementary material for: FOXO3a-driven miRNA signatures suppresses VEGF-A/NRP1 signaling and breast cancer metastasis
Source: Oncogene. 2020 Dec 1;40(4):777–90. doi: 10.1038/s41388-020-01562-y (PMC7843418; doi:10.1038/s41388-020-01562-y)
Supplement: Supplementary file 2 — Supplementary Table 1-5 [file 41388_2020_1562_MOESM2_ESM.docx]

**Supplementary Table 1. Analysis of the correlation between the expression of FOXO3a in breast cancer and its clinicopathological parameters.**

| Characteristics | Number of patients (n=100) | FOXO3a expression | | *P*-value |
| --- | --- | --- | --- | --- |
|  |  | Low (n=59) | High (n=41) |  |
| Age | | | | |
| <50 (years) | 40 | 22 | 18 | 0.5067 |
| >50 (years) | 60 | 37 | 23 |  |
| Histological grade | | | | |
| 1 | 21 | 7 | 14 | 0.0268 |
| 2 | 50 | 33 | 17 |  |
| 3 | 29 | 19 | 10 |  |
| Stage | | | | |
| I | 10 | 2 | 8 | 0.0211 |
| II | 72 | 44 | 28 |  |
| III-IV | 18 | 12 | 5 |  |
| Lymph node metastasis | | | | |
| Negative | 53 | 23 | 30 | <0.01 |
| Positive | 47 | 36 | 11 |  |
| Molecular subtype | | | | |
| ER+ | 51 | 20 | 31 | <0.01 |
| HER2+ | 19 | 12 | 7 |  |
| TNBC | 30 | 27 | 3 |  |

**Supplementary Table 2. Analysis of the correlation between the expression of VEGF-A/NRP1 in breast cancer and its clinicopathological parameters.**

| Characteristics | Number of patients (n=100) | VEGF-A expression | | *P*-value | NRP1 expression | | *P*-value |
| --- | --- | --- | --- | --- | --- | --- | --- |
|  |  | Low (n=38) | High (n=62) |  | Low (n=40) | High (n=60) |  |
| Age | | | | | | | |
| <50 (years) | 40 | 18 | 22 | 0.1647 | 16 | 24 | 1.000 |
| >50 (years) | 60 | 20 | 40 |  | 24 | 36 |  |
| Histological grade | | | | | | | |
| 1 | 21 | 10 | 11 | 0.3255 | 9 | 12 | 0.1083 |
| 2 | 50 | 20 | 30 |  | 24 | 26 |  |
| 3 | 29 | 8 | 21 |  | 7 | 22 |  |
| Stage | | | | | | | |
| I | 10 | 6 | 4 | 0.0383 | 8 | 2 | 0.017 |
| II | 72 | 26 | 46 |  | 24 | 48 |  |
| III-IV | 18 | 6 | 12 |  | 8 | 10 |  |
| Lymph node metastasis | | | | | | | |
| Negative | 53 | 32 | 21 | <0.01 | 27 | 26 | 0.0177 |
| Positive | 47 | 6 | 41 |  | 13 | 34 |  |
| Molecular subtype | | | | | | | |
| ER+ | 51 | 26 | 25 | 0.0237 | 26 | 25 | 0.0224 |
| Her2+ | 19 | 5 | 14 |  | 8 | 11 |  |
| TNBC | 30 | 7 | 23 |  | 6 | 24 |  |

**Supplementary Table 3. siRNA sequences used in this study.**

| Gene | siRNA Sequence |
| --- | --- |
| Control shRNA | GCTTCGCGCCGTAGTCTTA |
| FOXO3a shRNA-1 | GCTCACTTCGGACTCACTTAG |
| FOXO3a shRNA-2 | GGAACGTGATGCTTCGCAATG |

**Supplementary Table 4. Primers used in this study.**

| Gene | Sequence (5’-3’) |
| --- | --- |
| qRT-PCR primer | |
| FOXO3a-F | GCGTGCCCTACTTCAAGGATAAG |
| FOXO3a-R | GACCCGCATGAATCGACTATG |
| E-cadherin-F | TGCCCAGAAAATGAAAAAGG |
| E-cadherin-R | GTGTATGTGGCAATGCGTTC |
| Vimentin-F | AGTCCACTGAGTACCGGAGAC |
| Vimentin-R | GGTTCCTTTAAGGGCATCCAC |
| N-Cadherin-F | ACAGTGGCCACCTACAAAGG |
| N-Cadherin-R | CCGAGATGGGGTTGATAATG |
| NRP1-F | CATTGCTCGTTCCCCTCCTT |
| NRP1-R | TGTTTCTGGACCCGTTGGAG |
| GAPDH-F | CACCCAGAAGACTGTGGATGG |
| GAPDH-R | GTCTACATGGCAACTGTGAGG |
| TGF-β1-F | CAATTCCTGGCGATACCTCAG |
| TGF-β1-R | AGATAACCACTCTGGCGAGTC |
| TGF-β2-F | CAGCACACTCGATATGGACCA |
| TGF-β2-R | GTTGTAGATGGAAATCACCTCCG |
| VEGF-A-F | AGCCTTGCCTTGCTGCTCTA |
| VEGF-A-R | GTGCTGGCCTTGGTGAGG |
| VEGF-C-F | GAGGAGCAGTTACGGTCTGTG |
| VEGF-C-R | TCCTTTCCTTAGCTGACACTTGT |
| VEGF-D-F | TCCCATCGGTCCACTAGGTTT |
| VEGF-D-R | AGGGCTGCACTGAGTTCTTTG |
| PDGF-A-F | GCAAGACCAGGACGGTCATTT |
| PDGF-A-R | GGCACTTGACACTGCTCGT |
| PDGF-B-F | GGAGTCGAGTCGGAAAGCTC |
| PDGF-B-R | GTTCCGCGAGATCTGGAACA |
| PDGF-C-F | ATTCACAGCCCAAGGTTTCCT |
| PDGF-C-R | GGGTCTTCAAGCCCAAATCTTT |
| PDGF-D-F | CCCAGGAATTACTCGGTCAA |
| PDGF-D-R | ACAGCCACAATTTCCTCCAC |
| TNF-α-F | TCCTTCAGACACCCTCAACC |
| TNF-α-R | AGGCCCCAGTTTGAATTCTT |
| EGF-F | GGTCTTGCTGTGGACTGGAT |
| EGF-R | CTGCTACAGCAAATGGGTGA |
| HGF-F | GCCTGAAAGATATCCCGACA |
| HGF-R | GCCATTCCCACGATAACAAT |
| IGF-1-F | TCACCTTCACCAGCTCTGC |
| IGF-1-R | TGGTAGATGGGGGCTGATAC |
| IGF-2-F | AGACCCTTTGCGGTGGAGA |
| IGF-2-R | GGAAACATCTCGCTCGGACT |
| ChIP-PCR primers | |
| MiR-29b-2 site1-F | TGGAATAATACCTGCCCCATACG |
| MiR-29b-2 site1-R | TGGTTCCTGGCACTAACAGT |
| MiR-29b-2 site2-F | TCCTGGCCCAGGTGAGA |
| MiR-29b-2 site2-R | CTTTGTGTACTGTGAAATAC |
| Control site-F | AGAGGCCTGAAAGGAAGCTA |
| Control site-R | TCCCATCTCCTTACTGCATCAT |
| MiR-338 site-F | CAGCCCCAGACTCCATCAAG |
| MiR-338 site-R | GAAGGCTTCCTCTCCACTGC |
| Control site-F | CTTGCCCTGCCAGTGTCTG |
| Control site-R | GTGAGGCTAGGTCTCCCCAT |

**Supplementary Table 5. Antibodies used in this study.**

| **Antibody name** | **Manufacturer** | **Catalog no.** | **Working concentration** |
| --- | --- | --- | --- |
| FOXO3a | Cell Signaling Technology | #2497 | WB, 1:1000 |
| FOXO3a | Abcam | # ab12162 | IHC, 1:200  CHIP, 1:100 |
| VEGF-A | Abcam | # ab1316 | WB, 1:1000  IHC, 1:200 |
| NRP-1 | Abcam | #ab81321 | WB, 1:1000  IHC, 1:200 |
| E-Cadherin | Cell Signaling Technology | #3195 | WB, 1:1000 |
| N-Cadherin | Cell Signaling Technology | #13116 | WB, 1:1000 |
| Vimentin | Cell Signaling Technology | #5741 | WB, 1:1000 |
| VEGFR1 | Cell Signaling Technology | #2893 | WB, 1:1000 |
| VEGFR2 | Cell Signaling Technology | #9698 | WB, 1:1000 |
| AKT | Cell Signaling Technology | #4691 | WB, 1:1000 |
| p-AKT | Cell Signaling Technology | #4051 | WB, 1:1000 |
| ERK | Cell Signaling Technology | #4696 | WB, 1:1000 |
| p-ERK | Cell Signaling Technology | #4370 | WB, 1:1000 |
| β-actin | Cell Signaling Technology | #3700 | WB, 1:2000 |
