# Supplementary material for: FOXO3a-driven miRNA signatures suppresses VEGF-A/NRP1 signaling and breast cancer metastasis
Source: Oncogene. 2020 Dec 1;40(4):777–90. doi: 10.1038/s41388-020-01562-y (PMC7843418; doi:10.1038/s41388-020-01562-y)
Supplement: Supplementary file 3 — Supplementary Experimental Procedures [file 41388_2020_1562_MOESM3_ESM.docx]

**Supplemental Experimental Procedures**

**Wound-healing assay**

Cells were seeded into six-well plates at 5×10^5^ cells per well and allowed to grow to 90-95% confluence. A linear scratch was created using a sterile pipette. The cells were washed with PBS, and cultured with serum-free medium. Images were taken using a Leisa microscope with a 10x phase lens and the distance migrated was observed at 0, and 24 h after wounding.

**Transwell assay**

Cell invasion was evaluated by Matrigel-coated Transwell and Transwell inserts (BD Biosciences, San Diego, CA, USA). In total, 3 × 10^4^ cells suspended in 200 µL of serum-free medium were added to the upper chamber. The lower chambers were filled with the normal culture medium. The cells were incubated for 24 h at 37 °C. After incubation, the cells on the upper surface were removed, and the cells on the lower surface were fixed and stained with 0.1% crystal violet. The number of invaded cells was counted under a microscope in five predetermined fields for each membrane at ×200 magnification.

**ELISA assay**

Culture medium was collected and secreted VEGF-A was detected with the human VEGF-A ELISA kit (R&D Systems, Minneapolis, MN, USA) following the manufacturer’s instructions. ELISA plates were read using Lab Systems Multiscan EX Microplate reader at 450 nm with wavelength correction at 540 nm. The VEGF-A secretion was quantified using standard curve analysis within the linear range of 16 pg/ml to 2000 pg/ml.

**Chromatin immunoprecipitation (ChIP)**

ChIP was performed using SimpleChIP® Plus Enzymatic Chromatin IP Kit (Cell Signaling Technology, Cat. No 9005) following the manufacturer’s protocol. Briefly, cells were fixed with 1% formaldehyde for 10 min at RT. Next, the cells were washed twice with PBS at 4 °C, collected and resuspended in ice-cold lysis buffer and lysed on ice for 30 min. Cells were sonicated five times for 5 s to solubilize and shear cross-linked DNA. The chromatin (25 μg) was immunoprecipitated for 12 h with 2 μg of anti-FOXO3a antibody or IgG. After incubation, Protein G magnetic beads were then washed sequentially for 5 min with the following buffers: ChIP Buffer I for one time and ChIP Buffer II for two times. The immune complexes were eluted with elution buffer. After RNase A and proteinase K treatments and reversal of cross-linking, DNA was obtained by phenol and phenol/chloroform extractions. PCR amplifications of the precipitated DNA were carried out with oligos to FHRE-1 and FHRE-2 sequences of miR-29b-2 promoter and FHRE sequence of miR-338 promoter. The percentage of chromatin-bound recovered DNA was quantified against DNA input. Primers used for the amplification of the precipitated DNA are listed in supplementary Table S4.

**Dual-luciferase reporter assays**

Wild-type miR-29b-2 promoter region containing FOXO3a-binding sites (FHRE-1, FHRE-2) or mutant of miR-29b-2 promoter region, Wild-type miR-338 promoter region containing FOXO3a-binding site (FHRE) or mutant of miR-338 promoter region, were subcloned into pGL3 vector, respectively. All constructs were verified by sequencing. The 3’-untranslated regions (UTR) of VEGF-A, containing the predicted binding sites of miR-29b-2, and the 3’-UTR of NRP1, containing the predicted binding sites of miR-338, were amplified by PCR from genomic DNA and inserted into the luciferase reporter vector pmirGLO (Promega, Madison, WI, USA). By using these vectors as templates, we also generated mutant vectors with point mutations in the miR-29b-2-binding sites, or miR-338-binding sites using a QuikChange Site-Directed Mutagenesis kit (Stratagene, La Jolla, CA, USA). For the luciferase reporter assay, cells were seeded in 24-well plates in triplicate for 24 h and transfected with luciferase reporter constructs and pRL-TK Renilla luciferase. Cells were harvested and luciferase activity was measured 48 h later with a Dual-Luciferase Reporter Assay system (Promega) according to the manufacturer’s instruction. The Renilla luciferase activities were used as an internal control for transfection efficiency.

**Immunohistochemical assay**

Immunohistochemical assay was performed on formalin-fixed, paraffin-embedded sections of clinical breast cancer tissues. Briefly, the sections were deparaffinized in xylene, rehydrated with graded alcohol, and then boiled in 0.01 M citrate buffer (pH 6.0) for 20 min with an autoclave. Hydrogen peroxide (0.3%) was applied to block endogenous peroxide activity, and the sections were incubated with normal goat serum to reduce nonspecific binding. Tissue sections were incubated with the anti-FOXO3a antibody, anti-VEGF-A antibody, or anti-NRP1 antibody at 4 °C overnight. After incubation with the secondary antibody for 60 min, specimens were incubated with H_2_O_2_-diaminobenzidine until the desired stain intensity was developed. Sections were then counterstained with haematoxylin, dehydrated and mounted.

FOXO3a, VEGF-A and NRP1 protein expression were assessed by a semiquantitative immunoreactivity score. Staining intensity was scored as follows: negative (score 0), weak (score 1), moderate (score 2), and strong (score 3). Percentage scores were assigned as 1, 1-25%; 2, 26-50%; 3, 51-75%; and 4, 76-100%. The scores of each tumor sample were multiplied to give a final score of 0-12, and the tumors were finally determined as negative (−), score 0; lower expression (+), score ≤ 4; moderate expression (++), score 5-8; and high expression (+++), score ≥ 9. All immunohistochemical staining were evaluated and scored by at least two independent pathologists. The cutoff score was chosen based on a measure of heterogeneity using the log-rank test statistical analysis with respect to overall survival. Receiver operating curve (ROC) was used to determine the optimal cutoff score based on progression end point for FOXO3a, VEGF-A, and NRP1 expression. An optimal cutoff score was identified: a staining index of six or greater was used to define tumors of high expression, and five or lower for low expression.
